# Dynamical detection of network communities

**DOI:** 10.1038/srep25570

**Published:** 2016-05-09

**Authors:** Marcos G. Quiles, Elbert E. N. Macau, Nicolás Rubido

**Affiliations:** 1 Universidade Federal de São Paulo (Unifesp), Department of Science and Technology (DCT), 12247-014, São José dos Campos, SP, Brazil; 2 Laboratório Associado de Computação e Matemática Aplicada, Instituto Nacional de Pesquisas Espaciais, 12227-010, São José dos Campos, SP, Brazil; 3 Universidad de la República, Instituto de Física Facultad de Ciencias, Iguá 4225, 11400 Montevideo, Uruguay; 4 University of Aberdeen, King’s College, Institute for Complex Systems and Mathematical Biology, SUPA, AB24 3UE Aberdeen, United Kingdom

## Abstract

A prominent feature of complex networks is the appearance of communities, also known as modular structures. Specifically, communities are groups of nodes that are densely connected among each other but connect sparsely with others. However, detecting communities in networks is so far a major challenge, in particular, when networks evolve in time. Here, we propose a change in the community detection approach. It underlies in defining an intrinsic dynamic for the nodes of the network as interacting particles (based on diffusive equations of motion and on the topological properties of the network) that results in a fast convergence of the particle system into clustered patterns. The resulting patterns correspond to the communities of the network. Since our detection of communities is constructed from a dynamical process, it is able to analyse time-varying networks straightforwardly. Moreover, for static networks, our numerical experiments show that our approach achieves similar results as the methodologies currently recognized as the most efficient ones. Also, since our approach defines an N-body problem, it allows for efficient numerical implementations using parallel computations that increase its speed performance.

A remarkable feature observed in several complex networks is the presence of *communities*, namely, modular structures^1^,^2^,^3^,^4^, as it is observed, for example, on the Internet^5^, metabolic networks^6^,^7^, financial time-series^8^, or even in networks representing quantum systems^9^. Communities are groups of densely connected nodes within a network, while connections between nodes belonging to different communities are proportionally sparser. They characterize highly interactive local areas in a network, hence, their identification is important to understand the formation, growth mechanisms, and key structures of a network^10^,^11^. Moreover, the structure of communities shows similarities in regards to the characteristics of the nodes that compose them^4^,^12^. Thus, through the identification of communities we obtain fundamental information about the network characteristics.

Recently, various mechanism have been proposed for the emergence of communities^13^,^14^,^15^,^16^,^17^, which also derive the heavy-tail degree-distribution and high clustering commonly observed in real-world networks. Nevertheless, detecting communities in any observed network is still an extensive task. Let us take the simplest case of community detection: dividing a network into two parts of equal size such that the number of links connecting these two parts is minimal. This is already a complex task since the computational time to resolve it is non-polynomial, i.e., it is a *NP*-Complete problem^2^. In general, real networks may consist of an arbitrary number of communities, with several sizes, and hierarchical structures within themselves (namely, a community composed by other sub-communities)^4^, or even having soft^17^ or fuzzy^18^ communities (namely, nodes belonging partially to various communities), hence, the problem is even harder. Consequently, and given the importance and complexity of the community detection problem, several models have been proposed^18^,^19^,^20^,^21^,^22^,^23^,^24^,^25^,^26^,^27^,^28^,^29^,^30^,^31^,^32^,^33^,^34^,^35^,^36^,^37^,^38^,^39^,^40^. However, to the best of our knowledge, a method that efficiently detects these broad community scenarios and gives a physical interpretation for its process, is still missing.

Furthermore, real-world networks are usually time-varying, with sizes and structures that evolve continuously, complicating the community detection even further. For example, if we take a social network such as *Facebook*, new users (nodes) are added or removed daily and new friendships (links) are formed or eliminated. Similarly, an ecological network can change its trophic or symbiotic interactions, namely, the relationship between predators and preys or the intra-species interactions due to predations or competitions. Although several models for community detection in time-varying networks have been proposed, most are based on a static view-point of the network^4^, neglecting its intrinsic evolution. Specifically, these models work as follows. A static snapshot of the network at time *t* is obtained and the communities of the snapshot are detected by some algorithm. After the network changes, another snapshot is taken at time *t* + *δ* and the algorithm is reapplied. Thus, the network structures previously glimpsed are disregarded, as well as the community evolution from time *t* to *t* + *δ*.

Here, we propose a change in the community detection approach. We consider the nodes of the network as particles obeying a particular dynamics that promptly converges to clustered patterns, namely, the network communities. As a result, our approach makes a fast and optimal community detection, in particular, for time-varying networks. Moreover, is numerically efficient, since *N*-body problems allow for parallel computations, and is adjustable, since the choice of dynamics for the particles is flexible. This allows to conceive different algorithms which can be tailored to suit different data-sets, increase computational speed (i.e., convergence to the clustered patterns) or improve cluster separation (i.e., communities distinguishability).

Specifically, our approach associates the nodes of a network, e.g., Fig. 1(a), to an spatially distributed system of interacting particles, e.g., Fig. 1(b), hence, it introduces a physical interpretation to the detection of communities in networks. We choose the interaction between the particles to be either attractive (for nodes in the network that are adjacent, i.e., a link exists that connects them) or repulsive (for nodes in the network that are non-adjacent). The functional form for the interactions is chosen such that the system quickly achieves a clustered state, namely, the equilibrium one [Fig. 1(e)], where different particle clusters correspond to different network communities. This functional form is set following the general idea behind diffusive dynamical systems, where a potential function defines the particle dynamics so that the system evolves towards an asymptotically stable equilibrium. Hence, our approach is mathematically tractable within the dynamical system’s framework and solves elegantly the topological problems of community detection for any network, either static or time-varying. Also, by defining an *N*-body problem, it allows for efficient numerical implementations with parallel computations that increase its speed performance. In particular, we find that without parallel computations, our implementation has an 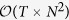 performance, *N* being the size of the network and *T* the number of iterations (see Supplementary Material for performance details).

## Results

### Model: complex networks as interacting particles

Let us consider a complex network 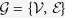, where 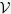 [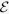] is the set of nodes [edges], for which we assign a set of particles in a *D*-dimensional space. We set *D* = 3 and start by placing the particles randomly, although neither the dimensionality of the space nor the initial distribution of particles seems critical. Our empirical findings show that results are nearly invariant if *D* ≥ 3, hence, *D* = 3 is the numerically most efficient and graphically straightforward situation we can choose, and the community detection is based on the asymptotic state of the particle system, hence, close but randomly placed particles suffice. The *i*-th node in the network (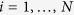) is then associated to a particle’s position, 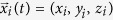, that evolves according to


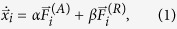


where 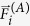 [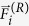] is the attractive [repulsive] interaction force that particle *i* is subject to due to the other particles (namely, the rest of the adjacent [non-adjacent] nodes) and *α* > 0 [*β* > 0] is the relative strength for the attractive [repulsive] force magnitude. These strengths constitute control parameters of our approach.

Let us now set the interaction between particles *i* and *j* such that, whenever nodes *i* and *j* in the network are connected, namely, the adjacency matrix *ij*-th entry is *A*_*ij*_ = 1, the corresponding particles feel a mutual attraction, 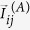. Contrary, if the adjacency matrix *A*_*ij*_ = 0, the corresponding particles feel a mutual repulsion, 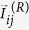. Consequently, the nodes that are [not] linked together correspond to particles that are [repelled] attracted to each other. These forces are designed so that the cumulative effect of all forces acting upon each particle (namely, 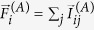 and 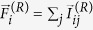), for optimally chosen values of *α* and *β*, drive the system of interacting particles to an asymptotic stable configuration in which the particles are attracted to different clusters. These clusters of particles are associated to the communities in the network, where particles that end in the same cluster identify a particular community in the network. Conceptually, we assume that if a community exists, the nodes within a community have a larger proportion of their links being shared within the community than the proportion of links connecting those nodes to other nodes outside their community. Hence, the corresponding particles within a community will have interactions that are more attractive than repulsive. Here, we consider the following interaction forces


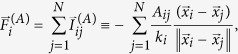


and


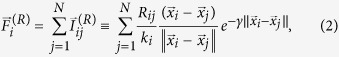


where *γ* > 0 is the characteristic decay rate for the repulsive interaction as a function of the distance between particles, namely, 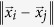, *A*_*ij*_ ≥ 0 is the adjacency matrix of the network, 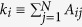 is the node’s degree, and 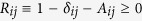 is the matrix of the absent links. We stress that other choices for the attractive and repulsive interactions are possible^31^, leading to a faster convergence or other clustered patterns (Supplementary Material), although the choice of placing an exponential term in the repulsion is done to guarantee the particles’ confinement. Without loss of generality, *γ* = 1 throughout our work.

The particular choice of interaction forces [Eq. (2)] we use makes our dynamical approach [Eq. (1)] a gradient system (Supplementary Material). Hence, it is a system that holds an attracting region, such that for any initial spatial distribution of particles close to the origin, the system converges to an equilibrium state. This final equilibrium-state corresponds to the network communities. In particular, to split these communities automatically, we use a clustering algorithm based on a centroids-seed approach, as explained in sec:methodsMethods. The clustering algorithm is similar to the *K*-means clustering algorithm, but with *K* varying dynamically. We note that force-directed algorithms^31^,^40^ share similarities with our gradient system, where an energy model is defined and its global minimum is sought. These algorithms also use attractive (repulsive) force between adjacent (non-adjacent) nodes, which cluster the nodes achieving a graphical layout where communities are observed^31^. However, our particle approach also includes the weighing factors *A*_*ij*_/*k*_*i*_ and *R*_*ij*_/*k*_*i*_ that correspond to the unbiased random-walk probabilities of a diffusive processes on the network^29^,^30^,^33^,^38^,^39^, namely, the transition probabilities for a random walker to diffuse from node *i* to *j* in a stochastic models. Since our numerical findings show that we overcome the problem of finding a local energy minima, we conjecture that the reason is due to the inclusion of these weighing factors into the particle dynamics.

### Numerical experiment: static networks

**Figure 2:**
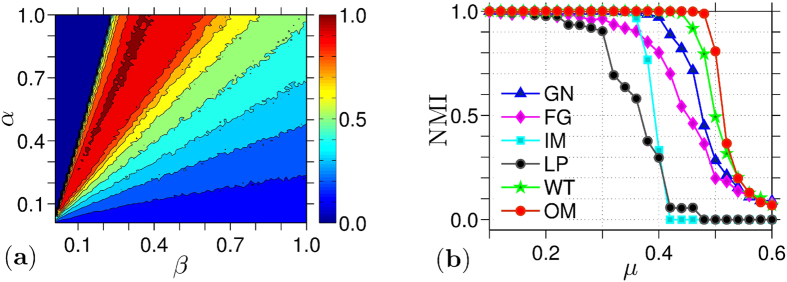
Community detection rates as a function of our parameters (left) and community distinguishability (right) in GN networks. The detection rates are measured by the normalized mutual information^2^ (NMI), which gives 1 [0] for a correct [an incorrect] detection (see Methods for details). Community distinguishability in Girvan-Newman (GN) networks^1^ is controlled by *μ*, a mixing parameter which blurs the community distinction as it is increased. Panel **(a)** shows the NMI (color code) that our algorithm gives as a function of the attractive and repulsive parameters, *α* and *β*, respectively, for a GN network with *N* = 128 nodes and *μ* = 0.45. Panel (**b**) shows the NMI for different state-of-the-art methods, namely, GN^1^, Fast Greedy^23^ (FG), Info MAP^30^ (IM), Label Propagation^25^ (LP), Walk Trap^29^ (WT), and our method (OM) [Eq. (1)] using *α* = 1 and *β* = 0.36, which achieves the best detection rate.

We use an explicit Euler scheme for the time discretization of the equations of motion [Eq. (1)] to have the fastest numerical evaluation, i.e., 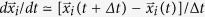 with Δ*t* = 1, and we use the *SNAP* package^41^ to implement our networks. The Euler scheme is always viable when the dynamics is a gradient system as convergence is then guaranteed. On the other hand, *α* and *β* in Eq. (1) are chosen from experiments with several networks. Our findings show that there is always a combination of values for these parameters that result in particle clusters. Namely, for each value of *α* one can find a value of *β* where communities are detected with a minimal error, as seen in Fig. 2(a), where the color code indicates the success rate that our model has (i.e., 0[1] corresponds to an unsuccessful[successful] detection) for a Girvan-Newman (GN) network^1^ of *N* = 128 nodes. The relationship between *α* and *β* is formally deduced in the Supplementary Material.

**Figure 3:**
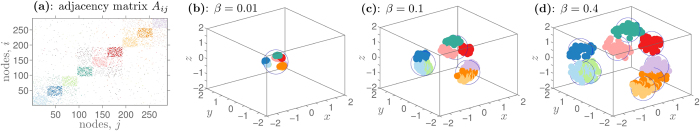
Hierarchical community detection of a network using the interacting particles’ approach. Our approach [Eqs. (1)–(2), 15] is applied to the network in panel (**a**), where the dots in the matrix represent a link in the network connecting node *i* (row) to *j* (column) and the color code is introduced to highlight the distinction between the 9 communities in the network. The network has *N* = 288 nodes, 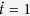, *μ*_1_ = 0.25 (for the micro-communities, which have sizes of 32 nodes), and *μ*_2_ = 0.08 (for the macro-communities, which have sizes of 96 nodes). Panels (**b**–**d**) show the final state of the particle system for different repulsion strengths (*β*) and fixed attraction strength (*α* = 1.0), starting from an arbitrary initial distribution of particles, as in Fig. 1(b). The community distinction emerges gradually and hierarchically as *β* is increased, which allows for soft community detection^17^.

We find that a successful community detection is possible for all static networks analysed when *α* = 1.0 and *β* ∈ (0.1, 0.4), as for example, is seen from Fig. 2(a). In general, if 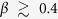, the repulsion is increased to a point where groups of particles are barely observed due to the influence of a strong repulsion. On the other hand, if 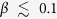, the attraction between particles overcomes repulsion causing all the particles or clusters to merge. These are the reasons why, as we vary *β* while holding *α* fixed, we detect a hierarchical structure of communities in the network from the resulting particle clusters. Figure 3 shows the particle’s asymptotic states for different values of *β* and fixed *α* = 1.0 on a network with 9 communities [Fig. 3(a)]. For small values of *β* (≃0.01), the 9 communities are merged into a single indistinguishable cluster of particles [Fig. 3(b)], however, as *β* is increased the particles start to cluster differently and communities are gradually detected, first 3 [Fig. 3(c)] and later 9 [Fig. 3(d)]. This parameter tuning provides a useful hierarchical detection of communities, showing the versatility of the particle approach, although, maintaining parameter robustness, namely, fine-tuning is generally unneeded (See the Supplementary Material for details on how to estimate *β*). In other words, we note that having a flexible choice for *β* allows us to have an algorithm which can detect soft^17^ or fuzzy^18^ communities as the parameter is tuned.

In order to evaluate the performance of our approach for community detection on general settings, we use the methodology considered in ref. 32. Particularly, we perform a set of experiments using the Girvan-Newman (GN)^1^ and Lancichinetti-Fortunato-Radicchi (LFR)^42^ benchmarks with the same parameters as in these references. On the other hand, in order to compare systematically our results on these benchmarks with other community detection methods [namely, Girvan-Newman^1^ (GN), Fast-Greedy^23^ (CNM), page-ranking^30^ (InfoMAP), label-propagation^25^ (PL), and Walk-trap^29^ (RAK) methods] we use the normalized mutual information (NMI)^2^, which measures the effectiveness that a community-detection method has to distinguish communities in any given network (see Methods for details).

The community structure of the networks in the GN and LFR benchmarks is controlled by a parameter known as the *mixing parameter*, *μ*. *μ* defines the proportion of links that a node in a community has connecting it to nodes from other communities. Meaning that, if *μ* = 0.0, the communities are completely isolated, namely, inter-community links are absent. If *μ* = 0.5, half of the node’s links are connections with other nodes in its own community (i.e., intra-community links), and the other half of the links are inter-communities links. Hence, as *μ* increases the distinction between communities is gradually lost, which constitutes a test for the robustness and reliability of the community-detection method.

**Figure 4:**
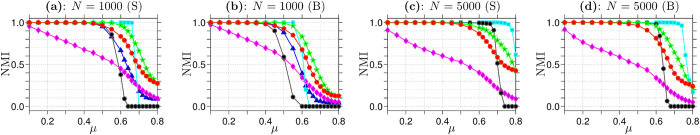
Community detection rates as a function of community distinguishability in LFR networks. The panels show the normalized mutual information^2^ (NMI) values as a function of the mixing parameter, *μ* (namely, the degree of community distinguishability), for the algorithms considered in Fig. 3(b) on Lancichinetti-Fortunato-Radicchi networks^32^. Four network scenarios are shown: *N* = 1000 and *N* = 5000 nodes with “small” (S) [panels (**a**,**c**)] and “big” (B) [panels (**b**,**d**)] communities (see Methods for details on the community sizes). Each point on the curves correspond to the average of the NMI value over 200 network realizations, excluding the GN analysis for *N* = 5000 because of its high computational cost. The symbols follow the labels set in Fig. 3(b) for each algorithm and the parameters for our algorithm are: *α* = 1 and *β* = 0.15 [panel (**a**)], 0.12 [panels (**b**,**c**)], and 0.09 [panel (**d**)].

For any method, as *μ* is increased from 0 to 1 and communities are gradually merged, the value of the NMI changes from 1 (i.e., all communities are properly detected) to 0 (i.e., no communities are detected). Ideally, the transition of the NMI values from 1 to 0 happens smoothly when 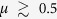, which corresponds to the situation where communities start being indistinguishable. In this sense, we see from Figs 2(b) and 4 that our approach detects communities effectively for both benchmarks, GN and LFR respectively, and even outperforms (on average) the other state-of-the-art community detection methods^1^,^23^,^25^,^26^,^29^,^30^ when *μ* is large. For example, we observe that our model can detect communities on the LFR benchmark to values up to 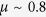 [Fig. 4(a)], which is a scenario where the community distinction is extremely subtle.

### Numerical experiment: time-varying network

**Figure 5:**
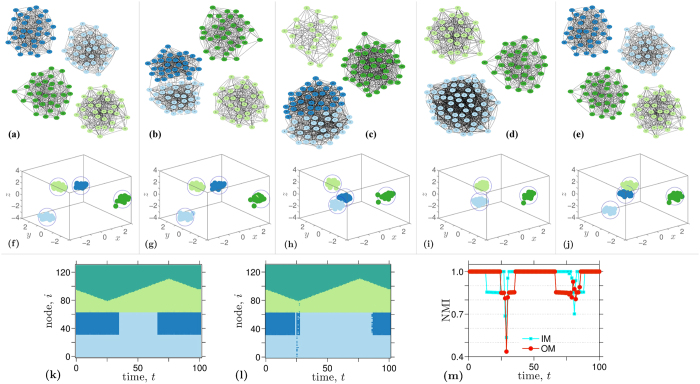
Evolution of our dynamical model of interacting particles for community detection on a time-varying complex network. Snapshots of the network evolution are shown in panels (**a**–**e**). The evolution starts with a network composed of 4 communities with 32 nodes each. From (**a**–**e**), using the methodology proposed in Granell, *et al.*^43^, the communities evolve dynamically by growing/shrinking and merging/splitting. Panels (**f**–**j**) depict the clustered particles’ steady-state of the networks in panels (**a**–**e**). Panel (**k**) illustrates in color code the evolution of the communities evolving in time and panel (**l**) illustrates the outcome of our approach, namely, the identification of the nodes belonging to one of the three or four communities present in panels **(a**–**e**). Panel (**m**) shows the NMI achieved by our model and by the Infomap^30^.

As an illustration of the efficiency of our approach in detecting the communities of time-varying networks, we show the results obtained for a particular scenario in Fig. 5. Using the methodology proposed in^43^, we start with a network of *N* = 128 nodes with 4 communities containing an even number of nodes (namely, 32), as shown in Fig. 5(a) (where inter-community links have been discarded on the graphical representation for the sake of clarity). Then, the communities evolve dynamically by growing/shrinking and merging/splitting. On a particular instant, after this modification on the network structure, two communities are effectively merged into a single community of 64 nodes, which leaves the network with a total of three communities [Fig. 5(d)]. As Fig. 5(f–j) show for each instant snapshot on Fig. 5(a–e) [*t* = 101, *t* = 103, *t* = 107, *t* = 110, and *t* = 120, respectively], the spatial configuration of particles after a few iterations rapidly converges into a new steady-state, that again, corresponds to the correct detection of the communities present in the modified network. Since the new community structure detected by our approach is obtained by running the algorithm from the former clustered state, the convergence speed is increased in comparison to that of a random initial condition (see Methods for details and implementation details).

### Numerical experiment: real-world network

**Figure 6:**
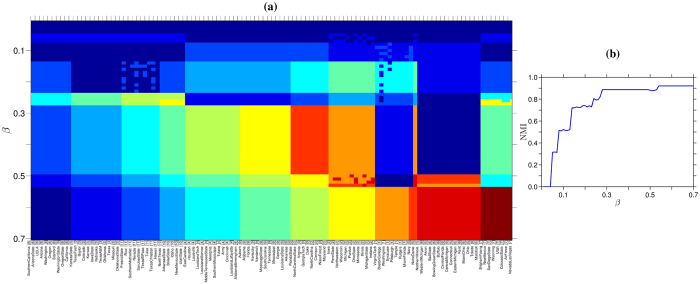
Hierarchical detection of communities on the American Football network. The color code in panel (**a**) corresponds to our hierarchical detection of the different communities that are observed in this network of *N* = 115 teams (nodes) where the football matches are the links. Each football team in this panel (horizontal axes) belongs to a community^22^, which is signalled by a number between 0 to 11. From top to bottom, as *β* is increased (i.e., the repulsive force strength), the communities that our approach detects start to split hierarchically, hence, more colors emerge. In particular, the NMI that we achieve on this network for different *β* values is shown in panel (**b**).

For the sake of completeness and to test the accuracy of our approach, we provide an experiments on a real-world social network. The experiment is conducted using the network of American football games introduced in ref. 22. This network contains 115 nodes, which represents the teams of the Division IA college games in the 2000 season. The links between nodes (teams) are the matches. The teams are split into twelve conferences of 8 to 12 teams. The matches between teams are more frequent between teams belonging to the same conferences, thus, we might expect the formation of communities. In our experiment, we perform a hierarchical detection of communities by varying the parameter *β* from 0.01 to 0.7. As it is shown in Fig. 6, the formation and the division of communities increases as the parameter *β* is increased. When *β* ≥ 0.55, the communities revealed by our method are compatible to those observed in the real division of the conferences^22^.

## Discussion

Our findings show that, treating a network as a set of interacting particles, where the force between particles is attractive [repulsive] when nodes are adjacent [non-adjacent] and is weighed by the random walk probability of transitioning between the nodes, allows to detect communities with high accuracy and low parameter sensitivity, outperforming several state-of-the-art community-detection algorithms. In summary, the main contributions from our approach are various. First, its dynamical nature. This means that, if a change in the network topology occurs, such as the inclusion or removal of a node or link, it is naturally interpreted as a perturbation in the particle system, thus reaching a new equilibrium state after a short transient. In this way, we avoid reapplying our approach when structural changes happen. On the contrary, most of the community detection methods are unable to deal with time-varying networks straightforwardly, since the algorithms must be reapplied every time a structural change is observed. Second, the adjustment of the interaction parameters allows us to detect communities hierarchically, which could also allow to identify networks with soft communities^17^. Third, the flexibility of our particle approach allows the choice of other functional forms for the interactions between particles, hence, designing different community-detection algorithms.

We note that, although the use of particle systems to solve a wide range of problems is well-known^19^ dating back to the use of molecular dynamics simulation techniques for hydrodynamic problems and celestial mechanics *N*-body problems, this particle approach is novel when it comes to community detection in networks. Furthermore, since *N*-body problems allow for parallel computations and we choose a particle dynamics that derives from a potential function, our approach allows for the design of numerically efficient and stable algorithms, namely, algorithms that require minimum floating point operations per iteration and allow for larger iteration time-steps.

## Methods

### Network benchmarks and the Normalized Mutual Information

We evaluate our methodology systematically following ref. 32 and using the normalized mutual information^2^ (NMI). Specifically, we perform a set of experiments taking networks that are considered benchmarks for testing community detection algorithms and evaluate the efficiency of our approach to detect communities on these networks by means of the resultant NMI value.

The *benchmarks* we choose are the Girvan-Newman (GN)^1^ networks and the Lancichinetti-Fortunato-Radicchi (LFR)^42^ networks, which are implemented using the same parameters as in these references. In particular, for the LFR networks the average degree was set to 20, the maximum degree to 50, the exponent of the degree distribution to −2.0, and the exponent of the community size distribution to −1.0. With these parameters, the following scenarios were considered: networks with 1000 nodes and community sizes varying from 10 to 50 nodes, which we name as small (S); networks with equal size but with communities varying from 20 to 100 nodes, which we name as big (B); and two scenarios more that follow the same range for the community size as the former two cases but with networks with 5000 nodes.

The effectiveness of the algorithms in detecting communities is quantified by the *normalized mutual information* (NMI) measure^2^. This measure is calculated from a confusion matrix **N**, where rows correspond to the expected community structure and the columns correspond to the obtained community structure. The NMI is then defined by


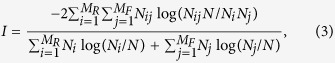


where *M*_*R*_ [*M*_*F*_] corresponds to the number of expected [found] communities, *N*_*ij*_ represents the number of nodes belonging to the real community *i* but clustered within community *j* according to the algorithm’s outcome, *N*_*i*_ [*N*_*j*_] defines the row [column] sum over *i* [*j*] of matrix **N**, and *N* represents the total number of nodes in the network.

### Community Detection Algorithm: centroids-seed approach

The particles in our approach [Eqs. (1)–(2), 15] self-organize into clusters after a short transient. This transient period is evaluated by analysing the instantaneous variations in the average of the repulsive interactions between particles, Δ*R*(*t*), where


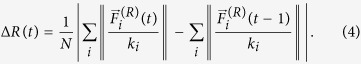


If Δ*R*(*t*) at time *t* is below a certain threshold *θ*_*r*_, an equilibrium state has been reached and the algorithm iterations can be stopped. This equilibrium state provides the community structure of the network for a given set of parameters (*α* and *β*).

In order to differentiate the particles belonging to different clusters automatically, after the transient, a centroids-seed approach is taken^34^. Namely, seeds are added randomly into the particle’s space with spatial position given by 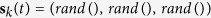, with 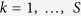, where *S* is the total number of seeds. These seeds 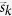 are used to identify the communities according to their membership. In order to identify the community that each node belongs to, a variable *y*_*i*_ is defined as the community label, i.e. if *y*_*i*_(*t*) = 1, it means that at time *t* node *i*, associated with particle 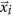, belongs to the community number 1, or in other words, is associated with the seed 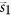.

Hence, the community assignment of each particle is done by evaluating the distance from the particle, e.g., 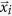, to all existing seeds, 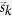, at time *t* by


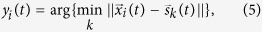


which means particle 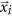 is always linked with its closest seed. We also calculate the quadratic error of each seed from


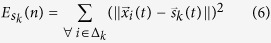


where Δ_*k*_ represents the set of particles associated with the seed s_*k*_ and |Δ_*k*_| is the number of particles in the set Δ_*k*_. Consequently, the error 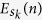 is somewhat the average quadratic distance of all particles within a cluster at iteration *n*. Then,

1. if any seed has error zero, 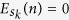, it means that this seed is isolated in the particle space, and it is associated with none or only one particle; consequently, the seed is removed;
2. if any seed has error greater than a threshold *θ*_*s*_, 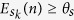, it means that this seed is associated with a highly heterogeneous cluster of particles, which indicates that a new cluster must be created. Thus, a new seed is inserted.

These conditions need the definition of a threshold *θ*_*s*_, which sets the maximum heterogeneity level allowed in each particle cluster. In particular, if any seed is removed or added, the particles are reassigned to the seeds [Eq. (5)]. Finally, the position of the seeds themselves are reassigned by


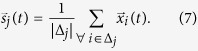


This process is repeated until the convergence of the seeds, which is observed when the variation of the errors of the seeds, 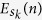, are stabilized. Namely, when 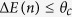, where


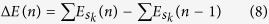


and *θ*_*c*_ we set constant at 10^−2^. From our numerical experiments, we observe that the number of steps (*n*) required to reach the stopping condition has a linear relationship to the number of detected communities, which we note is identical to the linear relationship found for the run-time reported for the Potts model^18^.

**Figure 7:**
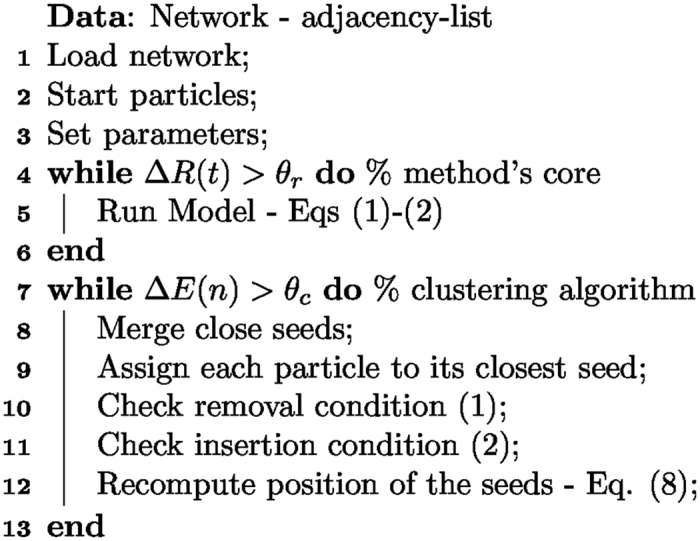
Community detection algorithm with the centroids-seed pseudo-code.

The overall algorithm is summarized in Fig. 7 and can be found in^44^. Throughout this work, we set the threshold parameters, namely, *θ*_*r*_ and *θ*_*s*_, to 10^−2^ and 0.5, respectively.

### Time-varying networks: algorithm implementation

In general, a time-varying network starts from an initial topology, namely, an initial network connectivity, and then it evolves its links according to some known or unknown function. Our method takes into account this initial network connectivity to calculate the equilibrium state of the associated particle system (where particles start from a random initial placement but close to the origin). Then, it evolves the particle systems from this initial equilibrium state at the same time as the links in the network are modified due to the network’s evolution. Also, if the network grows or shrinks as time evolves (i.e., *N* increases or decreases), particles are added close to the origin or removed. However, the network’s topology evolution is carried at a slower time-scale than the clustering dynamics between particles. Hence, we can think that at any time, we are pausing the network’s evolution and computing its communities, thus, retrieving a snapshot-like analysis. Nevertheless, we highlight the fact that our approach is applied continuously, contrary to the static-network snapshot method, hence, producing a real-time community-detection method that is unbiased.

The modified equations of motion for the particles are


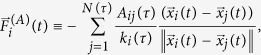



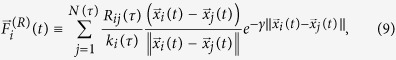


where 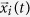 is the *i*-th particle position at time *t* and *A*_*ij*_(*τ*) [*R*_*ij*_(*τ*)] is the adjacency [complementary adjacency] matrix, that, at time *t*, has evolved *τ*. Similarly, the number of nodes in the network, *N*(*τ*), and their degree, 
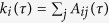
, which at time *t* has a value according to the evolution of the connectivity matrix *A*_*ij*_(*τ*). The time *τ* is the instantaneous time for the evolving topology, which in terms of *t*, is much slower. Namely, 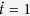 while 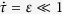, which is treating the evolution of the particle forces and the evolution of the connectivity as the decoupling of a dynamical system between its fast and slow dynamics.

**Figure 8:**
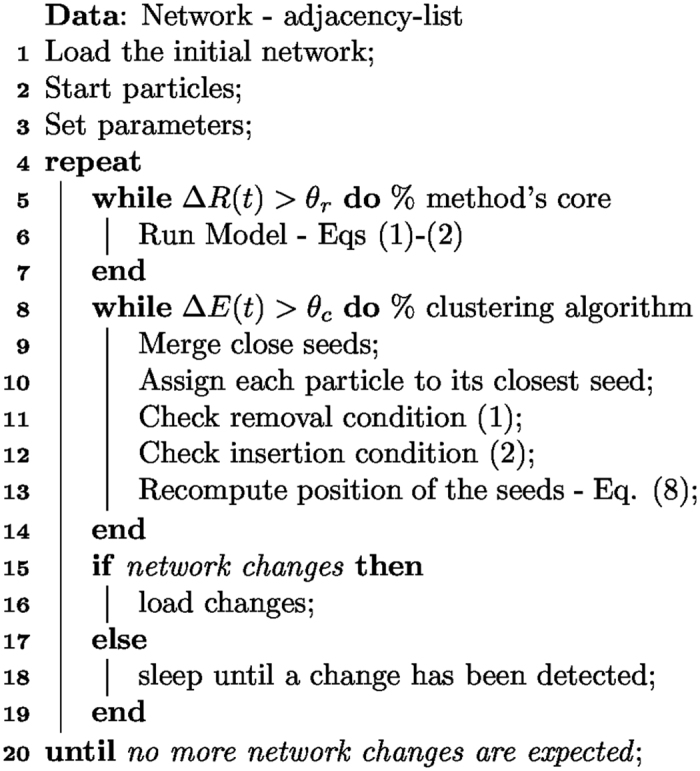
Community detection algorithm for time-varying networks.

The algorithm in Fig. 8 summarizes how the method is applied to time-varying networks. It is worth noting that, in contrast to Fig. 7, both the model’s core and the clustering routine do not start from a random initial condition, but from the condition built on the previous iteration. Thus, the number of steps necessary to reach a new equilibrium (position of the seeds) is lowered.

## Additional Information

**How to cite this article**: Quiles, M. G. *et al.* Dynamical detection of network communities. *Sci. Rep.* **6**, 25570; doi: 10.1038/srep25570 (2016).

## Supplementary Material

Supplementary Information

## Figures and Tables

**Figure 1:**
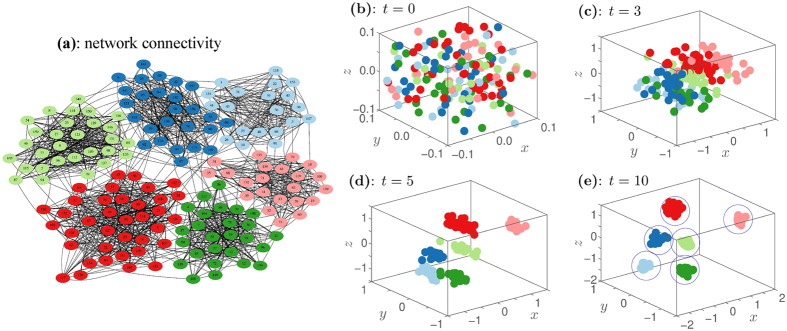
Evolution of our system of interacting particles for the detection of communities in a complex network. Panel (**a**) shows a network with 6 communities analyzed by our particle approach. We consider each node of the network as a particle. The particles interact with each other according to attractive (nodes that are connected in the network by a link) or repulsive (nodes in the network that are disconnected since a link is missing) forces. An arbitrary initial distribution (*t* = 0) of particles is shown in panel (**b**) that corresponds to the nodes in panel (**a**). The snapshots of the particles’ evolution at *t* = 3 [panel (**c**)], *t* = 5 [panel (**d**)], and *t* = 10 [panel (**e**)] show the fast convergence of the system to an equilibrium state and the resultant community detection from the particle clusters [encircled on panel (**e**)].
