# Supplementary material for: Dynamical detection of network communities
Source: Sci Rep. 2016 May 9;6:25570. doi: 10.1038/srep25570 (PMC4860646; doi:10.1038/srep25570)
Supplement: Supplementary Information [file srep25570-s1.pdf]

# Supplementary Material: Dynamical detection of network communities

**Marcos G. Quiles**[1], **Elbert E. N. Macau**[2], **and Nicolás Rubido**[3,4,*]

[1]Universidade Federal de São Paulo (Unifesp), Department of Science and Technology (DCT), 12247-014, São José dos Campos, SP, Brazil, [2]Laboratório Associado de Computação e Matemática Aplicada, Instituto Nacional de Pesquisas Espaciais (INPE), 12227-010, São José dos Campos, SP, Brazil, [3]Universidad de la República, Instituto de Física Facultad de Ciencias, Iguá 4225, 11400 Montevideo, Uruguay, [4]University of Aberdeen, King's College, Institute for Complex Systems and Mathematical Biology, SUPA, AB24 3UE Aberdeen, United Kingdom.
[*]Correspondence to: nrubido@fisica.edu.uy

## ABSTRACT

This is the Supplementary Material for the article "Dynamical detection of network communities".

## Dynamical system framework for community detection in complex networks

Our community detection approach is based on a set of coupled ordinary-differential-equations (ODEs). An ODE is[1,2]

$$\dot{\vec{x}}(t) = \frac{d\vec{x}}{dt} = \vec{F}\left[\vec{x}(t)\right], \tag{1}$$

where $\vec{x}(t)$ is the $D$-dimensional state vector ($\vec{x} : \mathbb{R} \to \mathbb{R}^D$) at time $t$ and $\vec{F}$ is the flow vector ($\vec{F} : \mathbb{R}^D \to \mathbb{R}^D$, which we assume to be smooth). The orbit of the system, or trajectory, is a particular solution of Eq. (1) for a given initial condition, i.e., the set of values given by $\{\vec{x}(t) \in \mathbb{R}^D \; \forall t \in I$ such that $\vec{x}(0) = \vec{x}_0\}$, $\vec{x}_0$ being the initial condition for the state vector and $I \subset \mathbb{R}$ the time interval where the solution is valid. We note that the flow vector in Eq. (1), besides the dependence on $\vec{x}$, may depend on particular parameters, namely, $\vec{F}(\vec{x};\vec{\alpha})$, with $\vec{\alpha} \in \mathbb{R}^P$, $P$ being the number of relevant parameters.

Given an ODE as in Eq. (1), a gradient system is such that

$$\dot{\vec{x}} = \vec{F}\left[\vec{x}(t)\right] = -\nabla V\left[\vec{x}(t)\right], \tag{2}$$

$\nabla$ being the gradient operator and $V$ being an scalar function, which is defined on an open set $\Upsilon$ containing $\vec{x}^*$ with $V : \Upsilon \to \mathbb{R}^+$ and $V(\vec{x}) > 0$ for $\vec{x} \neq \vec{x}^*$. Then, if $V$ is a Lyapunov function,[1,2] so that $\vec{x}^*$ is an isolated minimum of $V$, the solution of Eq. (2) is an asymptotically-stable equilibrium point. Thus, choosing a dynamical system for community detection that evolves according to Eq. (2), guarantees a globally attracting region. Moreover, each particular $V$ creates a different ratio of convergence and a different equilibrium state. Consequently, although our approach for community detection is tested using a particular choice of $V$, it is unrestricted to that particular $V$ and its convergence is improved when the steepness of $V$ is increased, as is also done in different energy models.[3]

In order to implement our approach to detect communities in networks, we choose a particular gradient function to define our particles' equations of motion, where each particle is associated with a node in the network and is embedded on a $D$-dimensional Euclidean space with position $\vec{x}_i$ for $i = 1,\ldots,N$. Moreover, its initial condition is randomly set. Thus, our particle system follows the equations of motion defined by

$$\dot{\vec{x}}_i = \vec{F}_i\left(\vec{x}_1,\ldots,\vec{x}_N\right) = -\nabla_i V, \quad \text{with} \quad V = \alpha \sum_{j=1}^{N} V_j^{(A)} + \beta \sum_{j=1}^{N} V_j^{(R)}, \tag{3}$$

where $\nabla_i$ is the gradient operator with respect to the space coordinates of particle $i$, i.e., $\nabla_i = (\frac{\partial}{\partial x_i}, \frac{\partial}{\partial y_i}, \frac{\partial}{\partial z_i})$, and the scalar potential function is divided into a sum of attracting, $V_j^{(A)}$, and repelling, $V_j^{(R)}$, non-negative scalar functions given by

$$V_j^{(A)} \equiv \frac{1}{2}\sum_{l=1}^{N} \frac{A_{jl}}{k_j} \left\|\vec{x}_j - \vec{x}_l\right\| \geq 0, \quad \text{and} \quad V_j^{(R)} \equiv \frac{1}{2\gamma}\sum_{l=1}^{N} \frac{R_{jl}}{k_j} e^{-\gamma\left\|\vec{x}_j - \vec{x}_l\right\|} \geq 0. \tag{4}$$

The reason for their non-negativity is because $\| \ \|$ is the Euclidean norm, $A_{jl} \geq 0$ is the adjacency matrix of the network (i.e., $A_{jl} = 1$ if there is a link connecting nodes $j$ and $l$ in the network or $A_{jl} = 0$ otherwise), $R_{jl} \equiv 1 - \delta_{jl} - A_{jl} \geq 0$ is the complement of the adjacency matrix, $\delta_{jl}$ being the Kronecker delta (i.e., $\delta_{jl} = 1$ if $j = l$ or $\delta_{jl} = 0$ otherwise), and $k_j \equiv \sum_l A_{jl}$ is the node's degree. These equations, namely, Eqs. (3) and (4), result in the equation of motion framework for our particle system and network community detection algorithm.

## Convexity of the attracting and repelling potential functions

We note that the arguments of $V_j^{(A)}$ and $V_j^{(R)}$ in Eq. (4) are mutually exclusive, namely, when one is positive the other one is null. The reason is that these functions depend on the network topology, namely, the symmetric adjacency matrix $A_{jl} = A_{lj}$, and the Euclidean distance between particles, namely, $\|\vec{x}_j - \vec{x}_l\| \geq 0$. Specifically, $V_j^{(A)}$ [$V_j^{(R)}$] depends on the existing [non-existing] links of the network of nodes, which are defined by $A_{jl}$. Thus, when $A_{jl} = 1$ (for $j \neq l$), $R_{jl} = 0$.

A convex function, $f : X \to \mathbb{R}$, fulfils $\forall x_1, x_2 \in X$, and $\forall \lambda \in [0,1]$ : $f[\lambda x_1 + (1-\lambda)x_2] \leq \lambda f(x_1) + (1-\lambda) f(x_2)$. Since the functions $V_j^{(A)}$ and $V_j^{(R)}$ in Eq. (4) depend on the distance between the particles, convexity is fulfilled only in particular conditions, for example, for $V_j^{(R)}$, when we consider the upper-half [lower-half] plane $\vec{x}_j - \vec{x}_l > 0$ [$\vec{x}_j - \vec{x}_l < 0$] alone. Moreover, since the potentials are a sum of distances between particles, convexity can be violated under other conditions as well. Nevertheless, we highlight that our numerical findings show that by including the Markov probabilities, namely, $A_{jl}/k_j$ and $R_{jl}/k_j$, in the arguments of the potential functions, the system still evolves towards an asymptotic state where cluster of particles are found for an attracting region close to the origin. In other words, although the convexity requirement for $V$ being a Lyapunov function is violated in a general scenario, the evidence we find points to the fact that the inclusion of the random walk probabilities avoids falling on a local minima of the potential function $V$.

## Differentiability of the potential function

The potential functions in Eq. (4) represent attractive and repulsive forces and the control parameters $\alpha$ and $\beta$ are the global magnitudes for the attractive and repulsive forces. From Eq. (3), we have that

$$\dot{\vec{x}}_i = -\alpha \left( \nabla_i \sum_{j=1}^{N} V_j^{(A)} \right) - \beta \left( \nabla_i \sum_{j=1}^{N} V_j^{(R)} \right) = -\frac{\alpha}{2} \left( \nabla_i \sum_{j=1}^{N} \sum_{l=1}^{N} \frac{A_{jl}}{k_j} \|\vec{x}_j - \vec{x}_l\| \right) - \frac{\beta}{2\gamma} \left( \nabla_i \sum_{j=1}^{N} \sum_{l=1}^{N} \frac{R_{jl}}{k_j} e^{-\gamma \|\vec{x}_j - \vec{x}_l\|} \right), \quad (5)$$

which defines the attractive and repulsive forces, namely, $\vec{F}_i^{(A)}$ and $\vec{F}_i^{(R)}$, to hold our equation of motion: $\dot{\vec{x}}_i = \alpha \vec{F}_i^{(A)} + \beta \vec{F}_i^{(R)}$.

We observe that applying the $x$-coordinate of the gradient to the distance between two particles holds

$$\frac{\partial \|\vec{x}_j - \vec{x}_l\|}{\partial x_i} = \frac{\partial}{\partial x_i} \sqrt{(x_j - x_l)^2 + (y_j - y_l)^2 + (z_j - z_l)^2} = \frac{(\delta_{ij} - \delta_{il})(x_j - x_l)}{\sqrt{(x_j - x_l)^2 + (y_j - y_l)^2 + (z_j - z_l)^2}}, \quad (6)$$

where, analogously, the remaining coordinates hold similar results. Hence,

$$\nabla_i \|\vec{x}_j - \vec{x}_l\| = (\delta_{ij} - \delta_{il}) \frac{(\vec{x}_j - \vec{x}_l)}{\|\vec{x}_j - \vec{x}_l\|}, \quad \text{and} \quad \nabla_i e^{-\gamma \|\vec{x}_i - \vec{x}_j\|} = -\gamma(\delta_{ij} - \delta_{il}) \frac{(\vec{x}_i - \vec{x}_j)}{\|\vec{x}_i - \vec{x}_j\|} e^{-\gamma \|\vec{x}_i - \vec{x}_j\|}. \quad (7)$$

We note that these equations are differentiable as long as their arguments, $\vec{x}_j - \vec{x}_l$, are different, namely, as long as $\vec{x}_j \neq \vec{x}_l$. This happens as long as particles are non-overlapping in space. Taking into account this consideration, the dynamical model for the particle's motion that follows from Eqs. (3)-(4) is given by

$$\vec{F}_i^{(A)} = -\sum_{j=1}^{N} \frac{A_{ij}}{k_i} \frac{(\vec{x}_i - \vec{x}_j)}{\|\vec{x}_i - \vec{x}_j\|}, \quad \text{and} \quad \vec{F}_i^{(R)} = \sum_{j=1}^{N} \frac{R_{ij}}{k_i} \frac{(\vec{x}_i - \vec{x}_j)}{\|\vec{x}_i - \vec{x}_j\|} e^{-\gamma \|\vec{x}_i - \vec{x}_j\|}, \quad \text{for } \vec{x}_i \neq \vec{x}_j. \quad (8)$$

## Dimensional analysis and relevant parameters

Our interacting particle model requires the determination of 3 parameters: $\alpha$, $\beta$, and $\gamma$. However, as we show here, there is only a single relevant parameter, namely, the ratio between the attractive and repulsive force strengths: $\eta \equiv \beta/\alpha$. The basis for our deduction is Buckingham $\pi$ theorem.

We start by noting that Eq. (3) can be written as

$$\frac{1}{\alpha} \frac{d\vec{x}_i}{dt} = \frac{1}{\alpha} \dot{\vec{x}}_i = \frac{\gamma}{\alpha \gamma} \frac{d\vec{x}_i}{dt} = \frac{1}{\alpha} \left( \alpha \vec{F}_i^{(A)} + \beta \vec{F}_i^{(R)} \right) = \vec{F}_i^{(A)} + \eta \vec{F}_i^{(R)} \equiv \gamma \frac{d\vec{x}_i}{d\tilde{t}}, \quad (9)$$

where we have introduced a dimensionless time $\bar{t} \equiv \alpha \gamma t$. The dimensions of $\alpha$ and $\gamma$ are those of $\vec{x}$ divided by time and the inverse of $\vec{x}$, respectively. Then, introducing a dimensionless vector $\vec{y}_i \equiv \gamma \vec{x}_i$ for all particles,

$$\frac{d\vec{y}}{d\bar{t}} = \vec{F}_i^{(A)} + \eta \vec{F}_i^{(R)}, \tag{10}$$

where after the change of variables, our interaction forces are written as

$$\vec{F}_i^{(A)} = -\sum_{j=1}^{N} \frac{A_{ij}}{k_i} \frac{(\vec{y}_i - \vec{y}_j)}{\|\vec{y}_i - \vec{y}_j\|}, \quad \text{and} \quad \vec{F}_i^{(R)} = \sum_{j=1}^{N} \frac{R_{ij}}{k_i} \frac{(\vec{y}_i - \vec{y}_j)}{\|\vec{y}_i - \vec{y}_j\|} e^{-\|\vec{y}_i - \vec{y}_j\|}. \tag{11}$$

These equations justify the use of $\gamma = 1$ in our work, since other values of $\gamma$ constitute a stretching or contraction of the unit of time, $\bar{t}$. Moreover, these equations also show the reason for setting $\alpha = 1$ and varying $\beta$, since this is effectively changing the setting to a single parameter, namely, going from the $2D$ $(\alpha, \beta)$-parameter space to the $1D$ $(1, \eta)$-parameter space.

### Equilibrium state

The equilibrium state is found when the system of particles achieves the steady-state, namely, when $\dot{\vec{x}}_i = 0$ for all particles. Hence, this is fulfilled when

$$\vec{F}_i^{(A)} + \eta \vec{F}_i^{(R)} = 0, \tag{12}$$

which corresponds to

$$\sum_{j=1}^{N} \frac{A_{ij}}{k_i} \frac{\left(\vec{x}_i^* - \vec{x}_j^*\right)}{\left\|\vec{x}_i^* - \vec{x}_j^*\right\|} = \frac{\beta}{\alpha} \sum_{j=1}^{N} \frac{R_{ij}}{k_i} \frac{\left(\vec{x}_i^* - \vec{x}_j^*\right)}{\left\|\vec{x}_i^* - \vec{x}_j^*\right\|} e^{-\gamma\|\vec{x}_i^* - \vec{x}_j^*\|}, \tag{13}$$

where $\vec{x}_i^*$ is the steady-state position for the $i$-th particle, corresponding to the potential $V$ minimum.

This is a transcendental equation[3] for the equilibrium state of the particle system. We note that the tuning of the parameters $\alpha$ and $\beta$ affect the equilibrium state as well as the modifying the connectivity of the network being analysed. This implies that finding a set of parameters that allows an accurate community detection requires a parameter tuning which depends on each particular network. However, we find that fine tuning of the parameters is generally unnecessary, meaning that parameter choices are robust. This allows to do a coarse search for parameter optimization and still find the optimal steady-state solution of the particle system.

## Numerical framework and algorithm performance

### Choosing the $\beta$ parameter

Although the model contains several parameters, most of them can be hold constant by using the values mentioned in the manuscript

- $\gamma = 1.0$
- $\alpha = 1.0$
- $\theta_c = 10^{-2}$
- $\theta_s = 0.5$
- $\theta_r = 10^{-2}$

The only remaining parameter that needs adjustment according to the network features and the desire community structure detection is the parameter controlling the repulsive force, namely, $\beta$, as it is also shown in Dimensional analysis. In general, by choosing small values for $\beta$, we find that the repulsion term [Eq. (14)] becomes small and the particle system tends to highlight macro-clusters, namely, giant communities, or even a single cluster grouping all particles. By increasing $\beta$, the repulsion becomes stronger and a division of the macro-clusters is observed. Interestingly, the sum of the error for the seeds during the centroid-seed method (i.e., $E(n) = \sum E_{sk}(n)$) in Eq. (8) of the manuscript) is an approximate indicator of the efficiency in the network's division by communities. The reason is twofold. On the one hand, the minima of $E(n)$ are associated to values of $\beta$ where the particles are well-confined in the clusters represented by the seeds. On the other hand, maxima values of $E(n)$ are

indicative that the particles represented by a seed are more separated than they should be, albeit insufficiently separated to split the cluster into more seeds.

In particular, Fig. 1 presents the analysis of $E(n)$ as a function of $\beta$ for a hierarchical network, which corresponds to the network shown in Fig. (3) of the manuscript. On the one hand, there are two clear minima in the $E(n)$ curve, one near $\beta = 0.05$ and another one near $\beta = 0.3$ (curve with triangles in Fig. 1). On the other hand, the first maxima of the NMI, achieved for this network using our particle approach, coincides with those two choices of $\beta$. Namely, the the macro-community detection, which is the curve with squares in Fig. 1, and the micro-community detection, which is the curve with circles in Fig. 1. Thus, by evaluating the evolution of $E(n)$, one can find target values for the $\beta$ parameter where a reasonable division of the network into communities is achieved.

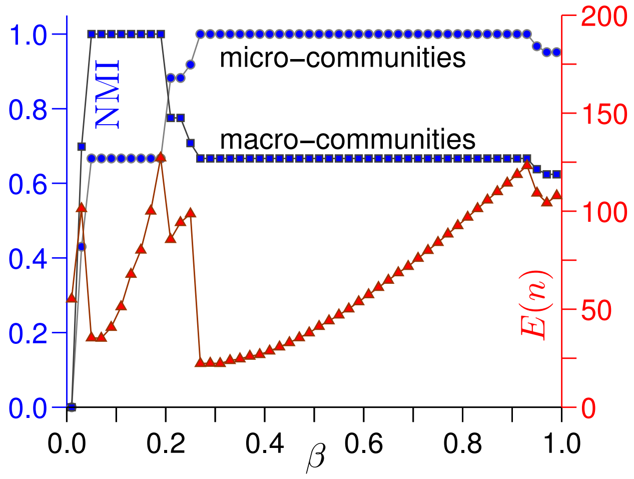


**Figure 1. Evaluation of the performance of the algorithm as the $\beta$ parameter (repulsive force strength) is increased.** The left vertical axes shows the normalized mutual information, NMI, as a measure of the efficiency of our approach to detect communities. In particular, there is a distinction in our algorithm's performance to detect macro- or micro-communities (curves with squares or circles, respectively). The right vertical axes shows the quadratic error, $E(n)$, of the centroid-seed approach we use to distinguish between different clusters of particles.

Moreover, since our model is intrinsically a dynamical model, the $\beta$ changes are taken into account naturally as a perturbation to the particle system. This means that, it is unnecessary to reapply the model from the beginning for each value of $\beta$. Instead, after the transient time for the particle system to settle in an equilibrium state for a given $\beta$ value, the following changes in $\beta$ can be observed as small perturbations to this equilibrium state for the particles and a new equilibrium state is reached only a few iterations afterwards. In other words, the changes in $\beta$ act as a bifurcation diagram, where at each stage that a community is unfolded into smaller communities a bifurcation has occurred.[1]

The former conclusions are valid for a broad range of networks. However, we note that for larger networks (i.e., $N \gg 10^4$) the $\theta_r$ parameter can also be increased to reduce the number of iterations that the algorithm takes to achieve the equilibrium state. Although, assigning a large value for $\theta_r$ can stop the particles' evolution prematurely leading to an ill-formation of particle clusters. This trade-off needs empirical adjustment for different classes of large networks.

## Computational time

The variation of the particle's positions is computed according to [Eq. (1) in the manuscript]

$$\dot{\vec{x}}_i = \alpha \vec{F}_i^{(A)} + \beta \vec{F}_i^{(R)}, \tag{14}$$

where $\vec{x}_i$ is the instantaneous position of the $i$-th particle associated to the network, with $i = 1, \ldots, N$.

In order to compute the former variation we need to evaluate the distance between each pair of nodes. Thus, each iteration of the model demands $\mathcal{O}(N^2)$ steps. The equilibrium is reached after some iterations, namely, $T$ when using an Euler scheme for the discretization of Eq. (14). Thus, the core computational-cost of our method is $\mathcal{O}(T \times N^2)$. However, we note that the number of iterations, $T$, varies depending on the network structure. Specifically, the larger is the mixing between the communities (i.e., more inter-connected communities), the more iterations are needed to reach the equilibrium state.

On the other hand, we have the clustering method. This method consists in finding the number of representative seeds (or communities), after the equilibrium state for the particles has been reached. The running time for each step of the clustering

method [see lines X-Y in Alg. (1) in the manuscript] is $\mathscr{O}(S \times N)$, where $S$ is the number of seeds (or detected communities). From our experiments, we observe that the clustering method demands $C$ steps, linearly related to $S$ by $C = \kappa S$ with $\kappa \simeq \mathscr{O}(1)$. Thus, the computational complexity of the clustering method is $\mathscr{O}(S^2 \times N)$.

Consequently, the total complexity of our approach for community detection is $\mathscr{O}(S^2 \times N + T \times N^2)$, considering both the particle dynamics and the clustering method. Since in general $N \gg S$, we have that the running time is $\mathscr{O}(T \times N^2)$.

Table 1, with its figure, depicts the CPU time needed to run the serial version of our code. The experiments were conducted in a 2.5 GHz Intel Core i5, with 8GB of RAM.

**Table 1. Computational time**. Numerical experiments on LFR Networks,[4] with network sizes $N = 10^4 : 10^5$, parameters $\mu = 0.1$, $\langle k \rangle = 50$, maximum degree $k = 100$, and community sizes from 500 to 1000. The values of the computational time $t$ (in seconds), the model iterations $T$, and the clustering method iterations $n$, are averages over 30 network realizations.

| $N$ | $t$ [$s$] | STD $t$ | $T$ | $n$ |
|---|---|---|---|---|
| 10000 | 489 | 124.46 | 143 | 118 |
| 20000 | 3203 | 860.56 | 172 | 135 |
| 30000 | 8005 | 2213.8 | 208 | 148 |
| 40000 | 14561 | 4081.6 | 217 | 157 |
| 50000 | 25518 | 6497.3 | 278 | 168 |
| 60000 | 39169 | 9143.4 | 312 | 181 |
| 70000 | 53448 | 12508 | 315 | 192 |
| 80000 | 71289 | 16308 | 332 | 203 |
| 90000 | 91732 | 20598 | 348 | 215 |
| 100000 | 114751 | 25382 | 392 | 223 |

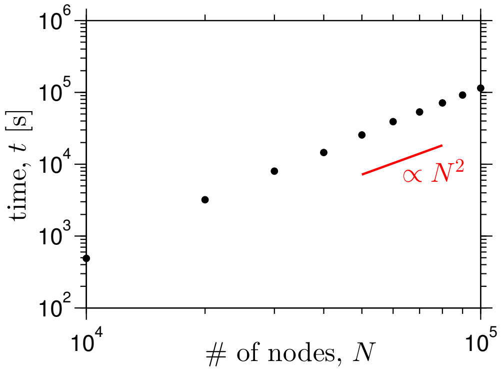


## References


1. Guckenheimer, J. and Holmes, P. *Nonlinear oscillations, dynamical systems, and bifurcations of vector fields* (Springer, Vol. 42, 1983).
2. Hirsch, M. W., Smale, S. and Devaney, R. L. *Differential Equations, Dynamical Systems, and an Introduction to Chaos* (Academic Press, Vol. 60, 2004).
3. Noack, A. Modularity clustering is force-directed layout. *Phys. Rev. E* **79**, 026102 (2009).
4. Lancichinetti, A. and Fortunato, S. Community detection algorithms: A comparative analysis. *Phys. Rev. E* **80**, 056117 (2009).
5. Leskovec, J. & Sosič, R. SNAP: Stanford Network and Analysis Platform. SNAP, (2014). Date of access: 11/05/2015. URL http://snap.stanford.edu/snap.
6. Quiles, M. G. Particle Community: A dynamical model for detecting communities in complex networks. GitHub, (2016). Date of access 28/01/2016. URL https://github.com/quiles/ParticleCommunity.
